# Practical Hints on the Management of Normal and Complicated Breech Cases

**Published:** 1908-10

**Authors:** Neilson Davie

**Affiliations:** M. B. Ch. B. (Glasgow), L. M. (Dublin.) Late Clinical Assistant, Coombe Hospital, Dublin; Assistant Medical Officer, Brecon and Radnor Asylum


### Practical Hints on the Management of Normal and Complicated Breech Cases.

### With a Few Notes on Their Diagnosis.

BY NEILSON DAVIE, M. B. Ch. B. (Glasgow), L. M. (Dublin.)
Late Clinical Assistant, Coombe Hospital, Dublin; Assistant Medical Officer, Brecon and Radnor Asylum.

(*The Hospital, Aug. 29, 1908.*)

THE chief dangers in breech cases are the extension of the arms and head, interference with the circulation of the cord, and delay in extracting the after-coming head, with the consequent risk of asphyxia to the child. Hence it is essential that a breech case should be diagnosticated early and suitable treatment be thus adopted in the first and second stages so as to minimise the risk of complications arising. If complications do arise they must be rectified as soon as possible. This must be done coolly and methodically, for once the accoucheur becomes flurried and excited the chances are that he rather hinders than expedites the delivery, and loses the child when it might have been saved. The incidence of breech cases is a little over 2 per cent. in labors with normal pelvis, and according to Herman they are as common again in cases where the pelvis is contracted. In the latter class of cases prolapse of the cord is apt to arise as a complication.

### DIAGNOSIS OF BREECH CASES.

In the diagnosis of breech cases the chief points to be noted are the following. On inspection the long axis of the uterus will be noticed to be in the long axis of the mother's abdomen. Palpation will reveal the presence of a hard, round, smooth, and solid tumor (the head) occupying the fundus. This tumor will be found to ballotte freely and to move independently of the body. In the pelvic grip the tumor is found to be well above the pelvic brim. It is larger, softer, and more irregular than the tumor in vertex presentation, and following it up by palpating along the dorsum of the child there is no interruption or depression to be discerned. The pelvic tumor does not ballotte freely, and moves with the body, thus contrasting with the fundal tumor. On auscultation the fetal heart is heard above the umbilicus to one or other side according to the position of the fetus.

The vaginal examination is most important in the diagnosis, and the following points can usually be made out in the early stage before a caput forms. If the membranes are intact there will be an irregular protrusion of the bag of membranes through the os. The presenting part will be found to be high up and not fixed. The trochanter can usually be felt. Next, if one can make out the bony triangle formed by the two ischial tuberosities and the sacrum of the fetus, there will be no hesitancy in deciding that the case is a breech. Within this triangle the finger may enter the anus, which must be distinguished from the mouth by the absence of the alveolar ridges and the tongue and by the presence of a sphincter grip and of meconium staining the finger. If the child is of the male sex the scrotum can usually be felt. If the perineum is much swollen, so that the parts cannot be felt distinctly, feel for a foot. See that there is no prolapse of the cord. Extreme care must be exercised in the examination and everything made out as gently as possible so as to avoid rupturing the membranes.

### TREATMENT.

In the first place the patient ought not to be allowed to walk about, but be kept in bed during the first stage. By keeping the patient in bed, straining and bearing down is reduced to a minimum, and hence the bag of membranes is better preserved. Remember that it is quite a calamity for the membranes to rupture early in the first stage. If they have burst, a powerful factor in the dilatation of the cervix is thereby lost. Avoid all unnecessary vaginal examinations, for the more made the greater the risk of sepsis supervening. Have the rectum well cleared out by a soap-and-water enema and also keep the bladder empty. Do not forget to keep up the patient's strength by giving her stimulating hot fluids—*e. q.*, beef tea, thin gruel, milk and tea, and the like. Have all preparations ready in case the child should be born in a state of asphyxia—*e. g.*, mucous extractor and two basins, one with hot water and the other with cold—also have a couple of towels soaking in a hot antiseptic solution.

When the cervix is fully dilated, if the membranes have not ruptured, rupture them. Then examine to see if the cord has prolapsed. Ensure that the bladder is empty. Next instruct your patient when and how to bear down, so that she may take as much advantage of her pains as possible. In an ordinary case allow the breech to remain intact, for if you bring a leg down the size of the advancing part is much reduced and it does not act so efficiently as a dilator of the soft parts. Avoid all traction on the legs or the breech, as this interferes with the natural flexion of the arms and head, and as a result they may become extended. Once the breech appears at the vulva put the patient in the cross-bed position with the hips well over the edge of the bed. In this position the accoucheur gets more room to work, and the fetal parts are not so liable to contamination. After the birth of the legs do not allow them to hang down, but hold them up and support them, otherwise extension of the arms and head may occur. Cover the feet, legs, and buttocks of child in a warm antiseptic towel which is ready prepared. This prevents contamination with the air, maternal parts, and the like. It also prevents reflex action causing attempts at respiration, and gives one a better grip of the fetus.

As soon as the umbilicus appears at the vulva insert the finger and draw down a loop of the cord and note if the pulsations are normal. If it is pulsating normally, and the child is making no attempts at respiration, which is noted by absence of movement of its abdomen, wait quietly and patiently, keeping the fingers on the cord all the time until the next pain comes on. Tell the mother to bear down as best she can when it comes on, and also get someone to press on the fundus from above downward and backward toward the sacrum, following the head down into the pelvis as it is being expelled. Usually this is quite sufficient to expel the fetus, and then, of course, the labor is completed as in an ordinary case. But at times, in spite of the greatest care, extension of the arms and head may occur.

### COMPLICATIONS AND THEIR TREATMENT.

The following are the chief complications which may arise in breech cases: (a) impacted breech; (b) interference with circulation of the cord: (c) extension of the arms and head; (d) prolapse of the cord; (e) separation of the placenta. I will make a few remarks on the first three only.

(a) In cases of impacted breech the first proceeding should be to pass the finger up into the child's groin. If at all possible insert both forefingers, one in each groin. Press them well against the abdomen, so as not to injure the femur, and pull downward and backward and then forward. If this method fails, bring down a foot. Failing this, pass a skein of wool as a fillet round the groin and pull. If this fails, forceps may be tried, but they are dangerous, as they are apt to slip and damage the maternal parts. If the child is alive and the breech cannot be got to move by any of the above methods, pubiotomy may be very successfully tried if the conjugate is above seven centimeters. If the child is dead, crush the breech.

(b) If the cord is pulsating feebly and intermittently this means that it is being pressed on, and artificial interference is necessary to save the child. Watch the abdomen of the child and note if there are any attempts at respiration, which may occur after the birth of the shoulders. If so, the head must be delivered immediately after the birth of the shoulders either artificially or naturally.

(c) If after the legs are born the patient gets a pain and the shoulders are not delivered, this is in all probability due to the extension of one or both arms above the head. Always free the posterior arm first, as there is no more room in the hollow of the sacrum. Pass up the hand which corresponds to the child's abdomen and flex the child's forearm down over the face into the vagina. Next free the anterior arm; if much difficulty is experienced reverse the position of the child so that the anterior arm becomes the posterior and then free it in the same way as the other. If the head is extended one method that may be tried to flex it is the following: Catch the feet in the left hand and put some traction on them in the axis of the mother and then in an upward direction. Pass the right hand along the child's abdomen and pass the forefinger into the mouth as far back as possible. Then exert traction on the lower jaw by means of the forefinger in the mouth. The direction of the traction is in a downward and backward direction. By the combined action of the two hands the head may be flexed and then delivered. Considerable help may be got by an assistant exerting pressure on the head above the pubis in such a way that flexion is promoted, whilst the traction is being exerted by the right forefinger. There are several other methods of delivery of the after-coming head which are too well known for enumeration in this article. In conclusion one may at an odd time have difficulty in delivering an after-coming head with the chin anterior. If the head is reversed it can often be quite easily delivered by the above-described method.

